# Dual Antiplatelet Therapy in Acute Branch Atheromatous Disease (BAD)‐Related Stroke: A Multicenter Propensity‐Matched Cohort Analysis

**DOI:** 10.1002/cns.71005

**Published:** 2026-06-30

**Authors:** Haizhou Hu, Shengde Li, Yu Zhang, Yuhui Sha, Yaping Zhou, Feng Feng, Yicheng Zhu, Lixin Zhou, Bin Peng, Jun Ni, BAD‐study investigators

**Affiliations:** ^1^ Department of Neurology State Key Laboratory of Complex Severe and Rare Diseases, Peking Union Medical College Hospital, Chinese Academy of Medical Sciences, and Peking Union Medical College Beijing China; ^2^ Department of Neurology Central Hospital Affiliated to Shandong First Medical University Jinan China; ^3^ Department of Neurology Xuanwu Jinan Hospital Jinan China; ^4^ Department of Radiology State Key Laboratory of Complex Severe and Rare Diseases, Peking Union Medical College Hospital, Chinese Academy of Medical Science and Peking Union Medical College Beijing China

**Keywords:** branch atheromatous disease, dual antiplatelet, ischemic stroke, outcome

## Abstract

**Aims:**

The optimal antiplatelet regimen for branch atheromatous disease (BAD)‐related stroke remains uncertain. This study aimed to compare the clinical outcomes of dual antiplatelet therapy (DAPT) vs. single antiplatelet therapy (SAPT) in these patients.

**Methods:**

From the multicenter prospective BAD‐study, we collected consecutive patients with BAD who received DAPT and SAPT. Propensity score matching (PSM) was used to balance baseline characteristics. The primary efficacy endpoint was an excellent outcome, defined as a modified Rankin Scale score of 0 to 1 at 90 days. The safety endpoint was bleeding events within 7 or 90 days.

**Results:**

A total of 449 patients were enrolled in the analysis, with a median age of 60 years and a median National Institutes of Health Stroke Scale score of 3 at admission. After PSM, there were 112 patients in the SAPT group and 171 patients in the DAPT group, with well‐balanced baseline characteristics. Excellent outcome occurred in 69.6% of the SAPT group and 79.5% of the DAPT group (odds ratio, 0.590; 95% confidence interval, 0.341 to 1.022; *p* = 0.059). No significant differences were observed in other efficacy outcomes between the two groups. In exploratory subgroup analysis, no significant treatment‐by‐subgroup interactions were observed, and after correction for multiple comparisons, no within‐subgroup differences remained statistically significant. No increased bleeding risk was observed in DAPT.

**Conclusion:**

In acute BAD‐related stroke, DAPT was safe but not statistically superior to SAPT for excellent functional outcome; however, its numerical trend toward benefit warrants further investigation.

## Graphical abstract

The study aimed to compare the clinical outcomes of dual antiplatelet therapy (DAPT) vs. single antiplatelet therapy (SAPT) in branch atheromatous disease (BAD)‐related stroke. DAPT was safe but not statistically superior to SAPT for excellent functional outcome; however, its numerical trend toward benefit warrants further investigation.

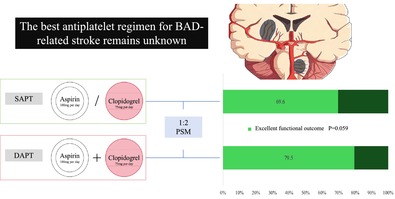

## 1 Introduction

Branch atheromatous disease (BAD), a pathological entity first proposed by Caplan in 1989 [1], is an important cause of subcortical infarction, accounting for 9.1%–20.4% of acute ischemic stroke (AIS) [2, 3, 4]. It is characterized by penetrating artery occlusion due to a clear atherosclerotic mechanism [1, 5]. Unlike large‐artery atherosclerosis, the parent large artery in BAD shows no evidence of severe stenosis. Furthermore, compared to small‐vessel disease arising from hypertensive lipohyalinosis, the proximal atherosclerotic lesion in BAD results in larger infarct volumes and a worse clinical course [2, 5]. BAD‐related stroke carries a high risk of early neurological deterioration (END) and is associated with poor outcomes [6, 7, 8]; however, no specific therapy is currently established [9]. Although several studies have investigated the use of argatroban or tirofiban in acute BAD‐related stroke, the efficacy of these agents remains uncertain [10, 11, 12]. Oral antiplatelet drugs such as aspirin and clopidogrel form the cornerstone of treatment for AIS [13], while the optimal antiplatelet strategy for BAD‐related stroke is unclear.

Dual antiplatelet therapy (DAPT) has been well‐established for secondary prevention in minor stroke or transient ischemic attack (TIA) [14, 15], though evidence regarding its effect on improving functional outcome remains limited. A sub‐analysis of the CHANCE trial reported a lower rate of 90‐day poor functional outcome (modified Rankin Scale [mRS] 2–6) in the DAPT group [16]. Similarly, a prospective study found that DAPT was associated with a lower incidence of END and a higher rate of 90‐day excellent functional outcome in minor stroke or TIA [17]. The ATAMIS trial demonstrated that DAPT was superior to aspirin alone in reducing END among patients with mild‐to‐moderate ischemic stroke, but it did not improve 90‐day functional outcome [18]. Notably, none of these studies detected an increased bleeding risk in DAPT [16, 17, 18]. However, these findings may not directly apply to BAD‐related stroke, which presents with a wide range of admission National Institutes of Health Stroke Scale (NIHSS) scores, spanning both minor and moderate neurological deficits [6, 19, 20].

Given that BAD is driven by focal atherosclerotic pathology at the ostium of penetrating arteries rather than intrinsic degenerative lipohyalinosis, the platelet activation and thrombus formation associated with plaque disruption may be more prominent than in a typical lacunar infarction [21]. DAPT, through complementary inhibition of thromboxane A2 synthesis and P2Y12‐mediated platelet aggregation, provides enhanced suppression of the multiple pathways involved in atherosclerosis‐related platelet reactivity [22]. This dual pathway blockade may confer greater antithrombotic protection in BAD than single antiplatelet therapy (SAPT).

Therefore, we conducted this multicenter, prospective study to evaluate the efficacy and safety of DAPT in acute BAD‐related stroke.

## 2 Methods

### 2.1 Patients and Design

We used data from the BAD‐study, which was a multi‐center, prospective cohort study investigating clinical, treatment, and prognosis characteristics of patients with BAD‐related stroke in 20 comprehensive stroke centers in China (NCT04973774) [23]. We enrolled patients aged 18 to 80 years with BAD‐related stroke undergoing oral antiplatelet therapy from June 2021 to June 2023. All patients met the radiological criteria of BAD‐related stroke: (1) Single deep infarction corresponding to the clinical symptoms, with typical lesion characteristics: For lenticulostriate artery (LSA) territory, a lesion spanning ≥ 3 consecutive axial diffusion‐weighted imaging (DWI) slices or exhibiting a comma shape on coronal imaging; for paramedian pontine artery (PPA) territory, a lesion extending from the deep to the ventral pons. (2) Absence of severe stenosis (≥ 50%) in the relevant parent artery. More detailed diagnostic criteria have been published elsewhere [23]. In this study, exclusion criteria were loss to follow‐up, lack of oral antiplatelet therapy, or the use of oral antiplatelet agents other than aspirin or clopidogrel.

### 2.2 Data Collection and Outcomes

Demographics, vascular risk factors, medication history, clinical features, radiologic data, and acute‐phase therapy were prospectively collected. We defined motor symptoms as the presence of new‐onset limb weakness, with or without accompanying sensory or other deficits. Acute‐phase therapy, recorded from stroke onset to 7 days after enrollment, included intravenous thrombolysis (IVT), antiplatelets, anticoagulants, and statins. The 90‐day mRS score was assessed via telephone interview or during an in‐person follow‐up visit.

In the SAPT group, patients received aspirin (100 mg per day) or clopidogrel (75 mg per day). In the DAPT group, patients received aspirin (100 mg per day) plus clopidogrel (75 mg per day) for 7 to 21 days, followed by aspirin or clopidogrel monotherapy. The choice between SAPT and DAPT, as well as other therapies, was at the discretion of the attending physician.

In this study, the primary efficacy endpoint was an excellent functional outcome, defined as mRS 0–1 at 90 days. Secondary efficacy endpoints included the proportion of 90‐day mRS 0–2, 90‐day mRS distribution, END, 7‐day NIHSS score, change in NIHSS score at 7 days, and vascular events (recurrent ischemic stroke, myocardial infarction, and intracranial hemorrhage) within 90 days. Safety endpoints were the incidence of bleeding and major bleeding events within 7 days and 90 days. END was defined as an increase of more than 4 points in NIHSS score or more than 1 point in NIHSS motor score from baseline within 7 days [6]. Major bleeding events were defined according to the PLATO criteria [24].

### 2.3 Statistical Analysis

Continuous variables were presented as mean ± SD or median [IQR], and compared with *t*‐test or Mann–Whitney U‐test, depending on their normality assessed by the Shapiro–Wilk test. Categorical variables were described using numbers (percentages) and compared between groups via Pearson's χ^2^ test or Fisher's exact test as appropriate. To minimize confounding, we used 1:2 propensity score matching (PSM) based on nearest‐neighbor matching incorporating all baseline characteristics with a caliper width of 0.05 (MatchIt package in R). Variables with a standardized mean difference (SMD) < 0.1 between groups were considered well‐balanced after PSM. To compare the functional outcomes of the SAPT and DAPT groups, we performed a binary logistic regression model to evaluate the 90‐day mRS 0–1, 90‐day mRS 0–2, and END, and an ordinal logistic regression model to evaluate the 90‐day mRS distribution. A generalized linear model was used to analyze the 7‐day NIHSS score and change in NIHSS score, and Cox regression analysis was used to analyze vascular and bleeding events.

To assess the robustness of the primary findings, we conducted sensitivity analysis for excellent functional outcome using the following approaches: (1) An unadjusted logistic regression model including all patients before PSM; (2) Multivariable logistic regression models adjusted for baseline variables based on selection threshold (*p* < 0.05 for model 1, and *p* < 0.1 for model 2) before PSM, to evaluate whether the result was sensitive to the covariate selection threshold; (3) Three clinically‐driven analyses using the same prespecified set of covariates (age, sex, hypertension, diabetes, hyperlipidemia, prior stroke, smoking, symptom type, baseline NIHSS score, onset‐to‐door time, culprit artery, intravenous thrombolysis, tirofiban use, argatroban use, and statins use) before PSM: A multivariable model 3, an inverse probability of treatment weighting (IPTW) model (weighted by propensity score [PS]), and a PS‐adjusted logistic regression model; and (4) A multivariable logistic regression model 4 within the PSM cohort, adjusted for the covariates in model 3.

We also explored the heterogeneity of treatment effect size on excellent functional outcome by test for interaction within the following subgroups: Age (< 60 or ≥ 60 years), sex (female or male), baseline NIHSS score (≤ 3 or > 3, ≤ 5 or > 5), symptom type (motor or other symptoms), occlusion site (LSA or PPA), IVT (yes or no), tirofiban (yes or no), argatroban (yes or no), and END (yes or no). A forest plot was used to better visualize the results of subgroup analysis (forestplot package in R). To account for multiple comparisons in the subgroup analysis, both within‐group and interaction *p*‐values were adjusted using the Benjamini–Hochberg false discovery rate (FDR) method.

Missing 7‐day NIHSS scores were excluded from the relevant analyses, as noted in Table 2. All analyses were performed by R software (version 4.4.1), with a two‐sided *p* < 0.05 considered statistically significant.

## 3 Results

**FIGURE 1:**
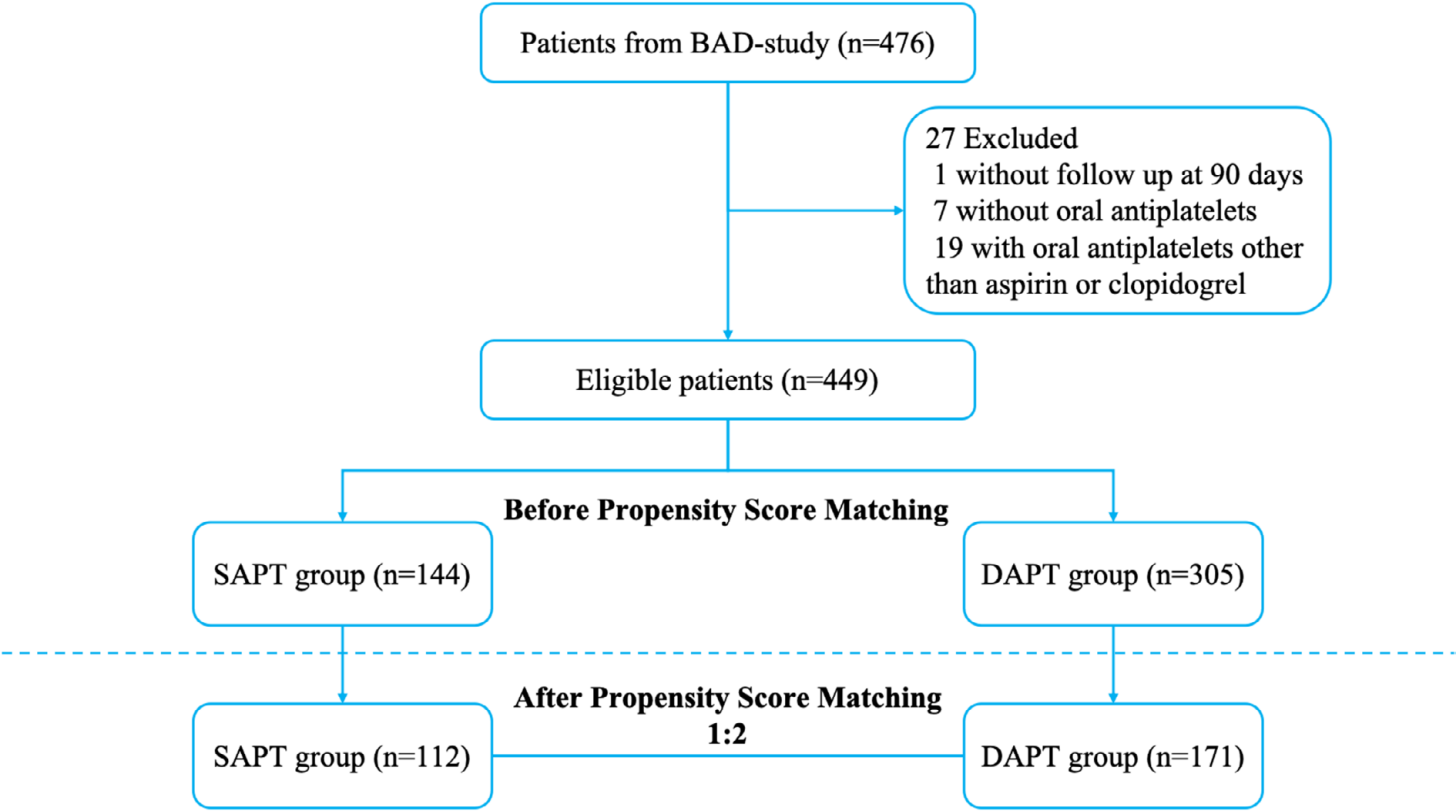
Study flowchart of patient enrollment. SAPT, single antiplatelet therapy; DAPT, dual antiplatelet therapy.

From the initial cohort of 476 patients in the BAD‐study, 27 patients were excluded (1 lost to follow‐up; 7 with no antiplatelet therapy; 19 with other antiplatelet agents). A total of 449 patients were enrolled in this analysis, including 144 who received SAPT and 305 who received DAPT (Figure 1). The median age was 60 (IQR: 53–67) years, with 315 (70.2%) male patients. The baseline NIHSS score was 3 (IQR: 2–6), and 72 (16.0%) patients received IVT. In the SAPT group, 129 (89.6%) patients received aspirin, with the remainder receiving clopidogrel. All patients in the DAPT group received both aspirin and clopidogrel.

The baseline characteristics before and after PSM are shown in Table 1. Before PSM, compared with the SAPT group, patients in the DAPT group were younger, had lower baseline NIHSS scores, and had a lower rate of hypertension but higher rates of smoking, motor symptoms, and treatment with IVT or tirofiban. After PSM, there were 112 patients in the SAPT group and 171 patients in the DAPT group, and all baseline variables were well balanced (all SMDs < 0.1, *p* > 0.05).

**TABLE 1:** Clinical characteristics of patients before and after propensity score match.

Variables	Before propensity score match					After propensity score match				
	Overall (*N* = 449)	SAPT (*N* = 144)	DAPT (*N* = 305)	*p*	SMD	Overall (*N* = 283)	SAPT (*N* = 112)	DAPT (*N* = 171)	*p*	SMD
Demographics										
Age	60.0 [53.0, 67.0]	63.0 [55.8, 68.0]	58.0 [52.0, 66.0]	**0.002**	**0.302**	60.0 [53.0, 67.0]	61.5 [53.8, 67.0]	60.0 [52.0, 67.5]	0.458	0.095
Sex				0.076	**0.178**				0.758	0.037
Male	315 (70.2)	93 (64.6)	222 (72.8)			200 (70.7)	78 (69.6)	122 (71.3)		
Female	134 (29.8)	51 (35.4)	83 (27.2)			83 (29.3)	34 (30.4)	49 (28.7)		
Vascular risk factors										
Hypertension	243 (54.1)	88 (61.1)	155 (50.8)	**0.041**	**0.208**	155 (54.8)	61 (54.5)	94 (55.0)	0.933	0.010
Diabetes	109 (24.3)	40 (27.8)	69 (22.6)	0.234	**0.119**	60 (21.2)	23 (20.5)	37 (21.6)	0.825	0.027
Hyperlipidemia	7 (1.6)	1 (0.7)	6 (2.0)	0.438	**0.111**	4 (1.4)	1 (0.9)	3 (1.8)	1.000	0.075
Coronary heart disease	30 (6.7)	10 (6.9)	20 (6.6)	0.878	0.015	19 (6.7)	7 (6.2)	12 (7.0)	0.801	0.031
Prior stroke	78 (17.4)	30 (20.8)	48 (15.7)	0.183	**0.132**	52 (18.4)	21 (18.8)	31 (18.1)	0.895	0.016
Smoking	184 (41.0)	47 (32.6)	137 (44.9)	**0.014**	**0.254**	98 (34.6)	39 (34.8)	59 (34.5)	0.956	0.007
Drinking	115 (25.6)	33 (22.9)	82 (26.9)	0.369	0.092	65 (23.0)	26 (23.2)	39 (22.8)	0.937	0.010
Family stroke	59 (13.1)	20 (13.9)	39 (12.8)	0.747	0.032	42 (14.8)	17 (15.2)	25 (14.6)	0.897	0.016
Medication history										
Antiplatelet agents	40 (8.9)	10 (6.9)	30 (9.8)	0.315	**0.104**	25 (8.8)	9 (8.0)	16 (9.4)	0.702	0.047
Anticoagulants	1 (0.2)	0 (0.0)	1 (0.3)	1.000	0.081	0 (0.0)	0 (0.0)	0 (0.0)	NA	< 0.001
Statins	26 (5.8)	5 (3.5)	21 (6.9)	0.148	**0.154**	15 (5.3)	5 (4.5)	10 (5.8)	0.611	0.063
Clinical features										
Symptom type				**0.004**	**0.282**				0.785	0.033
Motor symptoms	354 (78.8)	102 (70.8)	252 (82.6)			220 (77.7)	88 (78.6)	132 (77.2)		
Others	95 (21.2)	42 (29.2)	53 (17.4)			63 (22.3)	24 (21.4)	39 (22.8)		
NIHSS score at admission	3.0 [2.0, 6.0]	4.0 [2.0, 6.2]	3.0 [2.0, 6.0]	**0.026**	**0.208**	3.0 [2.0, 5.5]	3.0 [2.0, 6.0]	3.0 [2.0, 5.0]	0.805	0.025
SBP at admission	151.0 [138.0, 165.0]	152.0 [140.0, 169.2]	151.0 [137.0, 164.0]	0.120	**0.170**	151.0 [139.0, 166.5]	151.5 [138.0, 166.0]	151.0 [140.0, 167.0]	0.961	0.002
DBP at admission	90.0 [80.0, 100.0]	91.0 [81.0, 99.2]	90.0 [80.0, 100.0]	0.343	**0.103**	90.0 [81.0, 100.0]	91.0 [81.0, 99.0]	90.0 [81.0, 101.0]	0.980	< 0.001
Onset to door time	14.3 [6.0, 24.7]	17.2 [7.5, 24.8]	12.8 [5.0, 24.6]	0.051	**0.139**	15.0 [6.2, 25.0]	17.2 [7.0, 24.8]	14.2 [5.9, 25.2]	0.378	0.079
Acute‐phase therapy										
Intravenous thrombolysis	72 (16.0)	11 (7.6)	61 (20.0)	**0.001**	**0.364**	32 (11.3)	11 (9.8)	21 (12.3)	0.523	0.078
Antiplatelet agents' type										
Aspirin	434 (96.7)	129 (89.6)	305 (100.0)			272 (96.1)	101 (90.2)	171 (100.0)		
Clopidogrel	320 (71.3)	15 (10.4)	305 (100.0)			182 (64.3)	11 (9.8)	171 (100.0)		
Statins	421 (93.8)	131 (91.0)	290 (95.1)	0.093	**0.162**	267 (94.3)	106 (94.6)	161 (94.2)	0.861	0.021
Intravenous tirofiban	74 (16.5)	9 (6.2)	65 (21.3)	**< 0.001**	**0.448**	25 (8.8)	9 (8.0)	16 (9.4)	0.702	0.047
Intravenous argatroban	79 (17.6)	29 (20.1)	50 (16.4)	0.331	0.097	57 (20.1)	24 (21.4)	33 (19.3)	0.662	0.053
Infarction territory				0.348	0.095				0.952	0.007
Anterior	276 (61.5)	84 (58.3)	192 (63.0)			180 (63.6)	71 (63.4)	109 (63.4)		
Posterior	173 (38.5)	60 (41.7)	113 (37.0)			103 (36.4)	41 (36.6)	62 (36.3)		

*Note:* Bold values indicate statistical significance.

Abbreviations: SAPT, single antiplatelet therapy; DAPT, dual antiplatelet therapy; SMD, standardized mean difference; NIHSS, National Institutes of Health Stroke Scale; SBP, systolic blood pressure; DBP, diastolic blood pressure.

**FIGURE 2:**
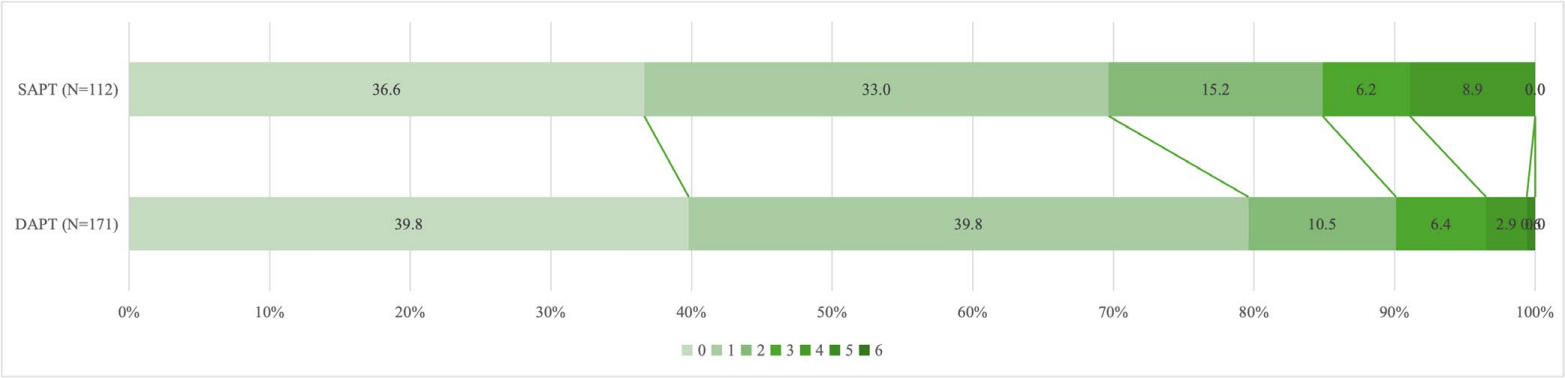
90‐day modified Rankin Scale (mRS) distribution after propensity score match. SAPT, single antiplatelet therapy; DAPT, dual antiplatelet therapy.

The comparison of efficacy endpoints between the two groups after PSM is shown in Table 2 and Figure 2. Excellent functional outcome occurred in 69.6% of the SAPT group and 79.5% of the DAPT group (odds ratio [OR], 0.590; 95% confidence interval [CI], 0.341 to 1.022; *p* = 0.059). There were no significant differences in secondary efficacy endpoints between SAPT and DAPT groups (all *p* > 0.05).

**TABLE 2:** Outcomes of patients with SAPT vs. DAPT after propensity score match.

Outcomes	SAPT (*N* = 112)	DAPT (*N* = 171)	Effect metric	Difference (95% CI)	P
90‐day mRS 0–1	78 (69.6)	136 (79.5)	OR^†^	0.590 (0.341–1.022)	0.059
90‐day mRS 0–2	95 (84.8)	154 (90.1)	OR^†^	0.617 (0.299–1.273)	0.188
Distribution of 90‐day mRS	1.0 [0.0, 2.0]	1.0 [0.0, 1.0]	cOR^‡^	1.354 (0.871–2.106)	0.178
Early neurological deterioration within 7 days	18 (16.1)	18 (10.5)	OR^†^	1.628 (0.803–3.301)	0.174
7‐day NIHSS score^a^	3.0 [1.0, 5.0]	3.0 [1.0, 5.0]	GMR^§^	0.016 (−0.054–0.086)	0.659
Change in NIHSS score at 7 days from baseline^a^	1.0 [0.0, 2.0]	0.0 [0.0, 1.0]	GMR^§^	−0.014 (−0.073–0.044)	0.629
Vascular events within 90 days	1 (0.9)	3 (1.8)	HR^||^	0 (0‐Inf)	0.999
Recurrent ischemic stroke within 90 days	1 (0.9)	2 (1.2)	HR^||^	0 (0‐Inf)	0.999
Bleeding within 7 days	1 (0.9)	0 (0.0)	HR^||^	Inf (0‐Inf)	0.999
Major bleeding within 7 days	1 (0.9)	0 (0.0)	HR^||^	Inf (0‐Inf)	0.999
Bleeding within 90 days	2 (1.8)	3 (1.8)	HR^||^	1.542 (0.217–10.945)	0.665
Major bleeding within 90 days	1 (0.9)	0 (0.0)	HR^||^	Inf (0‐Inf)	0.999

Abbreviations: SAPT, single antiplatelet therapy; DAPT, dual antiplatelet therapy; CI, confidence interval; OR, odds ratio; cOR, common odds ratio; GMR, geometric mean ratio; HR, hazard ratio; mRS, modified Rankin Scale; NIHSS, National Institutes of Health Stroke Scale; IVT, intravenous thrombolysis.

^†^ Calculated with binary regression analysis.

^‡^ Calculated with ordinal regression analysis.

^§^ The log (NIHSS+1) was analyzed using a generalized linear model.

^||^ Calculated with the Cox regression model.

^a^ Two missing data points in the DAPT group.

The sensitivity analyses for excellent functional outcome included the unadjusted model, multivariable models 1–3, IPTW model, PS‐adjusted model before PSM, and the post‐PSM multivariable model 4 (Table S1). Across all models, the direction of the observed trend consistently favored DAPT, with ORs ranging from 0.559 (post‐PSM multivariable model 4) to 0.704 (PS‐adjusted model), although none reached statistical significance (p range: 0.053 to 0.141).

**FIGURE 3:**
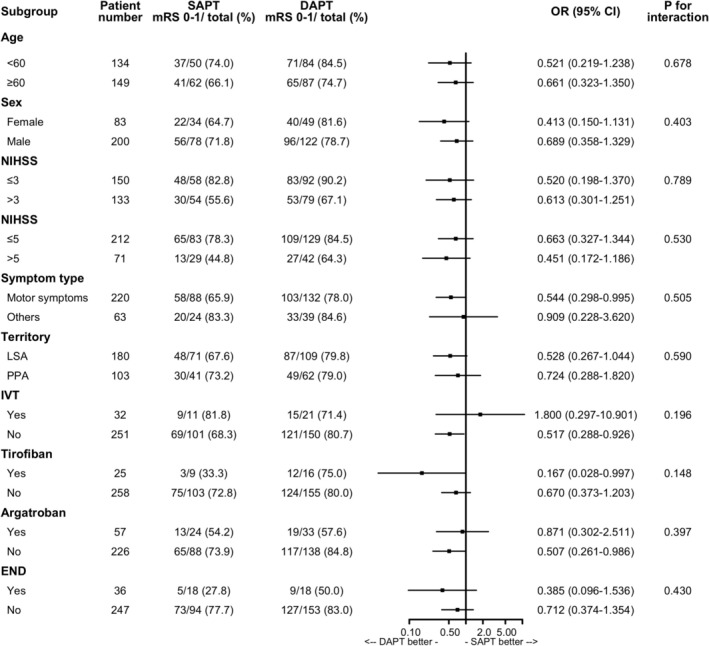
Subgroup analysis after propensity score match. SAPT, single antiplatelet therapy; DAPT, dual antiplatelet therapy; OR, odds ratio; CI, confidence interval; NIHSS, National Institutes of Health Stroke Scale; LSA, lenticulostriate artery; PPA, paramedian pontine artery; IVT, intravenous thrombolysis; END, early neurological deterioration.

In the exploratory subgroup analysis (Figure 3 and Table S2), no significant interaction was observed for any subgroup regarding the effect of SAPT vs. DAPT on 90‐day mRS 0–1 (all p_interaction_ > 0.05). Before correction for multiple testing, in patients with motor symptoms (OR, 0.544; 95% CI, 0.298 to 0.995; *p* = 0.048), those not receiving IVT (OR, 0.517; 95% CI, 0.288 to 0.926; *p* = 0.026), and those not receiving argatroban (OR, 0.507; 95% CI, 0.261 to 0.986; *p* = 0.045), the SAPT group had a lower proportion of achieving excellent functional outcome compared with the DAPT group. After applying FDR correction for multiple comparisons, none of the within‐subgroup differences remained statistically significant.

For safety endpoints assessed before and after PSM (Table S3 and Table 2), there was no significant difference in the incidence of any or major bleeding within 7 or 90 days (all *p* > 0.05).

## 4 Discussion

In this multicenter, prospective cohort study, we employed PSM to compare the efficacy and safety of DAPT vs. SAPT in BAD‐related stroke. Our analysis revealed that DAPT was associated with a trend toward better 90‐day functional outcome, although the difference did not reach statistical significance (*p* = 0.059). In exploratory subgroup analysis, no significant treatment‐by‐subgroup interactions were observed, and all nominal within‐subgroup associations became statistically non‐significant after correction for multiple comparisons. Notably, no increased risk of bleeding events was observed with DAPT, even though half of the patients had an admission NIHSS score > 3. These findings argue against routinely excluding those with relatively severe neurological deficits.

Given that the pathogenesis of BAD‐related stroke is driven by atherosclerosis rather than cardioembolism, antiplatelet therapy represents the mainstay of treatment [9]. As a more intensive regimen, DAPT may be associated with additional benefit by inhibiting multiple pathways of platelet activation, potentially reducing thrombus progression, which may contribute to better functional outcome. This hypothesis remained unconfirmed owing to the lack of statistical significance. Nevertheless, a numerical trend favoring DAPT was observed, with an approximately 10% higher rate of excellent functional outcome (79.5% vs. 69.6%). The negative results may be related to the relatively small sample size and the inclusion of unselected patients with BAD‐related stroke. Thus, large‐scale, randomized controlled trials RCTs with refined designs are warranted to definitively test this hypothesis.

In exploratory subgroup analysis, no statistically significant interactions were observed between treatment assignment and any subgroups. Before correction for multiple testing, the proportion of patients achieving an excellent functional outcome appeared nominally lower in the SAPT group among those with motor symptoms, those not receiving IVT, and those not receiving argatroban. However, after applying multiple testing correction, none of the within‐subgroup differences remained statistically significant. These findings indicate that the observed nominal differences are highly susceptible to chance and do not provide reliable evidence for differential treatment effects across subgroups. Accordingly, the subgroup patterns should be interpreted with extreme caution. In addition, the proportion of excellent functional outcome was lower in patients who received argatroban (56.1%) or tirofiban (60.0%) than in those who did not. This may be fundamentally explained by the observational nature of our cohort study. As non‐standard therapies reserved as rescue interventions, argatroban and tirofiban were initiated typically after neurological deterioration [25, 26, 27], which was inherently associated with poor outcomes [6, 8].

Bleeding is a primary concern with DAPT. In our cohort of BAD‐related stroke, the 90‐day incidence of bleeding events in the DAPT group was 1.8%, comparable to the 2.3% reported in the CHANCE trial for minor stroke and TIA [14]. This suggested a favorable safety profile of DAPT in BAD‐related stroke, even though only half of our patients met the CHANCE criteria (admission NIHSS score ≤ 3). The safety of DAPT in patients with a higher NIHSS score may be explained by the unique lesion characteristics of BAD‐related stroke [6, 8]. Typically, a higher NIHSS score correlated with larger infarct volume and thus increased risk of hemorrhagic transformation [28], while this relationship was altered in BAD. Occlusion of a deep penetrating artery commonly resulted in a relatively small infarction. However, due to the involvement of the pyramidal tracts, it may cause severe motor deficits (e.g., hemiplegia) that disproportionately elevate the NIHSS score [29]. Consequently, BAD‐related stroke may present with clinically significant deficits driven by a strategically located, small infarct core, which may not confer the same hemorrhagic risk as larger territorial infarction. Therefore, the NIHSS score alone should not be the sole determinant in choosing between SAPT and DAPT for BAD‐related stroke. Rather, clinical decisions should be guided by an individualized assessment that integrates factors such as infarct volume [28], cerebral small vessel disease (CSVD) burden, [30] and other patient‐specific risk profiles.

In summary, despite a negative primary endpoint, our real‐world study suggests a trend favoring DAPT for functional outcome in BAD‐related stroke, with a favorable safety profile regarding bleeding. Exploratory subgroup analyses revealed no significant interactions, and all nominal differences were non‐significant after correction for multiple comparisons. Our study suggests that the safety of DAPT supports reconsidering the automatic exclusion of individuals based solely on a relatively high NIHSS score.

Several limitations of this study should be acknowledged. First, the assignment of SAPT or DAPT was not randomized; although PSM was used to balance known confounders, residual confounding may persist. Second, the oral antiplatelet agents in our study were limited (aspirin and clopidogrel), except for cilostazol, which was promising for BAD‐related stroke [20]. But aspirin and clopidogrel had stronger evidence and better accessibility in ischemic stroke. In addition, the CYP2C19 genotype was not tested or recorded; given that 50%–60% of Asians carry a loss‐of‐function allele [31], the observed efficacy of DAPT in our cohort may be attenuated. Furthermore, DAPT duration varied from 7 to 21 days. While this reflects real‐world practice, shorter durations may bias results toward the null, making our findings conservative. Finally, the relatively small sample size restricted the statistical power, which may affect the interpretation of our results.

## 5 Conclusion

In this real‐world study of acute BAD‐related stroke, DAPT, although not statistically superior to SAPT for excellent functional outcome, was safe and associated with a numerical trend toward benefit, findings that warrant further investigation.

## Author Contributions

Haizhou Hu, Shengde Li, and Jun Ni conceived and designed the study. Haizhou Hu analyzed the data and wrote the first draft. Shengde Li, Yu Zhang, Yuhui Sha, and Yaping Zhou performed data collection and revised the manuscript. Feng Feng, Yicheng Zhu, Lixin Zhou, Bin Peng, and Jun Ni provided critical review and edited the manuscript. All authors read and approved the final version of the manuscript.

## Funding

This work was supported by the National High‐Level Hospital Clinical Research Funding (2022‐PUMCH‐D‐007); the Noncommunicable Chronic Diseases‐National Science and Technology Major Project (2023ZD0515700); and the Special Project on Clinical Scientific Research of the Medical and Health Technology Development and Research Center, National Health Commission (WKZX20205CZ0301).

## Disclosure

The authors have nothing to report.

## Ethics Statement

The study was approved by the ethics committee of Peking Union Medical College Hospital (No. I‐23PJ792), and written informed consent of all participants was obtained.

## Conflicts of Interest

The authors declare no conflicts of interest.

## Supporting information


**Table S1:** Sensitivity analysis for excellent functional outcome in patients with SAPT vs. DAPT.
**Table S2:** Subgroup analysis for excellent functional outcome in patients with SAPT vs. DAPT after propensity score match.
**Table S3:** Safety outcomes of patients with SAPT vs. DAPT before and after propensity score match.

## Data Availability

The data that support the findings of this study are available from the corresponding author upon reasonable request.
