# Supplementary material for: Dual Antiplatelet Therapy in Acute Branch Atheromatous Disease (BAD)‐Related Stroke: A Multicenter Propensity‐Matched Cohort Analysis
Source: CNS Neurosci Ther. 2026 Jun 30;32(7):e71005. doi: 10.1002/cns.71005 (PMC13317594; doi:10.1002/cns.71005)
Supplement: Supplementary file 1 — Table S1: Sensitivity analysis for excellent functional outcome in patients with SAPT vs. DAPT. Table S2: Subgroup analysis for excellent functional outcome in patients with SAPT vs. DAPT after propensity score match. Table S3: Safety outcomes of patients with SAPT vs. DAPT before and after propensity score match. [file CNS-32-e71005-s001.docx]

**Table S1. Sensitivity analysis for excellent functional outcome in patients with SAPT versus DAPT.**

| **Excellent functional outcome** | **OR (95% CI)** | **P** |
| --- | --- | --- |
| Before PSM (N=449) |  |  |
| Unadjusted model^a^ | 0.649 (0.420-1.008) | 0.053 |
| Multivariable model 1^b^ | 0.654 (0.391-1.095) | 0.105 |
| Multivariable model 2^c^ | 0.645 (0.385-1.082) | 0.096 |
| Multivariable model 3^d^ | 0.618 (0.359-1.063) | 0.081 |
| IPTW model^e^ | 0.608 (0.356-1.036) | 0.067 |
| PS-adjusted model^f^ | 0.704 (0.442-1.127) | 0.141 |
| After PSM (N=283) |  |  |
| Multivariable model 4^g^ | 0.559 (0.299-1.040) | 0.067 |

Abbreviations: SAPT, single antiplatelet therapy; DAPT, dual antiplatelet therapy; OR, odds ratio; CI, confidence interval; IPTW, inverse probability of treatment weighting; PS, propensity score; PSM, propensity score match.

^a^ All patients were included without adjusting for any variables.

^b^ Adjusted for baseline variables with P < 0.05 (age, hypertension, smoking, symptom type, admission NIHSS score, intravenous thrombolysis, and intravenous tirofiban).

^c^ Adjusted for baseline variables with P < 0.1 (age, sex, hypertension, smoking, symptom type, admission NIHSS score, onset-to-door time, intravenous thrombolysis, intravenous tirofiban, and statins therapy after admission).

^d^ Adjusted for baseline variables that may affect outcome (age, sex, hypertension, diabetes, hyperlipidemia, prior stroke, smoking, symptom type, baseline NIHSS score, onset-to-door time, intravenous thrombolysis, tirofiban use, argatroban use, statins use, and culprit artery).

^e^ Weighted by the same variables as multivariable model 3.

^f^ PS score was derived from the same variables as multivariable model 3.

^g^ Adjusted for the same variables as multivariable model 3.

**Table S2. Subgroup analysis for excellent functional outcome in patients with SAPT versus DAPT after propensity score match.**

|  | **Patient number** | SAPT mRS 0-1/ total (%) | DAPT mRS 0-1/ total (%) | **OR (95%CI)** | **P** | **FDR-**  **adjusted P** | **P for interaction** | **FDR-**  **adjusted P for interaction** |
| --- | --- | --- | --- | --- | --- | --- | --- | --- |
| **Age** |  |  |  |  |  |  |  |  |
| <60 | 134 | 37/50 (74.0) | 71/84 (84.5) | 0.521 (0.219-1.238) | 0.140 | 0.310 | 0.678 | 0.753 |
| ≥60 | 149 | 41/62 (66.1) | 65/87 (74.7) | 0.661 (0.323-1.350) | 0.256 | 0.356 |  |  |
| **Sex** |  |  |  |  |  |  |  |  |
| Female | 83 | 22/34 (64.7) | 40/49 (81.6) | 0.413 (0.150-1.131) | 0.085 | 0.283 | 0.403 | 0.738 |
| Male | 200 | 56/78 (71.8) | 96/122 (78.7) | 0.689 (0.358-1.329) | 0.267 | 0.356 |  |  |
| **NIHSS** |  |  |  |  |  |  |  |  |
| ≤3 | 150 | 48/58 (82.8) | 83/92 (90.2) | 0.520 (0.198-1.370) | 0.186 | 0.310 | 0.789 | 0.789 |
| >3 | 133 | 30/54 (55.6) | 53/79 (67.1) | 0.613 (0.301-1.251) | 0.179 | 0.310 |  |  |
| **NIHSS** |  |  |  |  |  |  |  |  |
| ≤5 | 212 | 65/83 (78.3) | 109/129 (84.5) | 0.663 (0.327-1.344) | 0.254 | 0.356 | 0.530 | 0.738 |
| >5 | 71 | 13/29 (44.8) | 27/42 (64.3) | 0.451 (0.172-1.186) | 0.107 | 0.306 |  |  |
| **Symptom type** |  |  |  |  |  |  |  |  |
| Motor symptoms | 220 | 58/88 (65.9) | 103/132 (78.0) | 0.544 (0.298-0.995) | 0.048 | 0.250 | 0.505 | 0.738 |
| Others | 63 | 20/24 (83.3) | 33/39 (84.6) | 0.909 (0.228-3.620) | 0.892 | 0.892 |  |  |
| **Territory** |  |  |  |  |  |  |  |  |
| LSA | 180 | 48/71 (67.6) | 87/109 (79.8) | 0.528 (0.267-1.044) | 0.066 | 0.264 | 0.590 | 0.738 |
| PPA | 103 | 30/41 (73.2) | 49/62 (79.0) | 0.724 (0.288-1.820) | 0.492 | 0.579 |  |  |
| **IVT** |  |  |  |  |  |  |  |  |
| Yes | 32 | 9/11 (81.8) | 15/21 (71.4) | 1.800 (0.297-10.901) | 0.522 | 0.580 | 0.196 | 0.738 |
| No | 251 | 69/101 (68.3) | 121/150 (80.7) | 0.517 (0.288-0.926) | 0.026 | 0.250 |  |  |
| **Tirofiban** |  |  |  |  |  |  |  |  |
| Yes | 25 | 3/9 (33.3) | 12/16 (75.0) | 0.167 (0.028-0.997) | 0.050 | 0.250 | 0.148 | 0.738 |
| No | 258 | 75/103 (72.8) | 124/155 (80.0) | 0.670 (0.373-1.203) | 0.180 | 0.310 |  |  |
| **Argatroban** |  |  |  |  |  |  |  |  |
| Yes | 57 | 13/24 (54.2) | 19/33 (57.6) | 0.871 (0.302-2.511) | 0.798 | 0.840 | 0.397 | 0.738 |
| No | 226 | 65/88 (73.9) | 117/138 (84.8) | 0.507 (0.261-0.986) | 0.045 | 0.250 |  |  |
| **END** |  |  |  |  |  |  |  |  |
| Yes | 36 | 5/18 (27.8) | 9/18 (50.0) | 0.385 (0.096-1.536) | 0.176 | 0.310 | 0.430 | 0.738 |
| No | 247 | 73/94 (77.7) | 127/153 (83.0) | 0.712 (0.374-1.354) | 0.300 | 0.375 |  |  |

SAPT, single antiplatelet therapy; DAPT, dual antiplatelet therapy; OR, odds ratio; CI, confidence interval; FDR, false discovery rate; NIHSS, National Institutes of Health Stroke Scale; LSA, lenticulostriate artery; PPA, paramedian pontine artery; IVT, intravenous thrombolysis; END, early neurological deterioration.

**Table S3. Safety outcomes of patients with SAPT versus DAPT before and after propensity score match.**

| **Safety outcomes** | **Before propensity score match** | | | | **After propensity score match** | | | |
| --- | --- | --- | --- | --- | --- | --- | --- | --- |
|  | **SAPT (N=144)** | **DAPT (N=305)** | **HR (95% CI)** | **P** | **SAPT (N=112)** | **DAPT (N=171)** | **HR (95% CI)** | **P** |
| Bleeding within 7-day | 1 (0.7) | 0 (0.0) | Inf (0-Inf) | 0.999 | 1 (0.9) | 0 (0.0) | Inf (0-Inf) | 0.999 |
| Major bleeding within 7-day | 1 (0.7) | 0 (0.0) | Inf (0-Inf) | 0.999 | 1 (0.9) | 0 (0.0) | Inf (0-Inf) | 0.999 |
| Bleeding within 90-day | 2 (1.4) | 3 (1.0) | 2.135 (0.301-15.156) | 0.448 | 2 (1.8) | 3 (1.8) | 1.542 (0.217-10.945) | 0.665 |
| Major bleeding within 90-day | 1 (0.7) | 0 (0.0) | Inf (0-Inf) | 0.999 | 1 (0.9) | 0 (0.0) | Inf (0-Inf) | 0.999 |

Abbreviations: SAPT, single antiplatelet therapy; DAPT, dual antiplatelet therapy; HR, hazard ratio; CI, confidence interval.
